# CldU sensitizes *BRCA2* reverse-mutated cells to PARP inhibitors

**DOI:** 10.3389/fonc.2025.1626301

**Published:** 2025-11-19

**Authors:** Nawel Zouggari, Camilla Trugenberger, Valentine Du Bois, Wenwen Wang, Giacomo G. Rossetti, Thanos D. Halazonetis, Intidhar Labidi-Galy

**Affiliations:** 1 Department of Medicine and Center of Translational Research in Onco-Hematology, Faculty of Medicine, University of Geneva, Geneva, Switzerland; 2 Department of Molecular and Cellular Biology, Faculty of Sciences, University of Geneva, Geneva, Switzerland; 3 Department of Oncology, Geneva University Hospitals, Geneva, Switzerland

**Keywords:** BRCA mutation, PARP inhibitor, resistance, reversion mutation, thymidine analogue, CldU, cancer

## Abstract

PARP inhibitors are widely used class of drugs for the treatment of homologous recombination deficient cancers, including *BRCA* mutated ones. These drugs led to substantial improvement in survival, particularly for patients with *BRCA* mutated tumors. However, many patients eventually develop resistance to PARP inhibitors, mainly due to *BRCA* reversion mutations. Overcoming resistance to PARP inhibitors is an unmet medical need. Recently, it has been shown that *BRCA*-deficient cells are hypersensitive to the thymidine analogue 5-chloro-2’-deoxyuridine (CldU), either alone or in combination with PARP inhibitors. In this study, we show, across multiple **BRCA*2* mutated cell lines, that CldU sensitizes PARP inhibitor-resistant cells to PARP inhibitors. This synergy was also present in cell lines with **BRCA*2* reversion mutations and was associated with high levels of DNA damage and arrest in S phase. This effect, which is specific to thymidine analogue CldU, may open new avenues for the treatment of *BRCA* mutated cancers resistant to PARP inhibitors.

## 1 Introduction

Poly(ADP-ribose) polymerase (PARP) inhibitors (PARPi) induce cell death by exploiting the absence of homologous recombination in cancer cells harboring mutations in the *BRCA1/BRCA2* genes (1). More precisely, cancer cells lacking the repair proteins BRCA1 and BRCA2 rely more heavily on PARP to repair their damaged DNA. Hence, inhibiting PARP leads to cell death as these cells are no longer able to repair the damage to their DNA. Studies have shown that loss of *BRCA2* leads to cells being 100 to 1000 times more sensitive to PARPi, this led to their exploitation in the clinic in the context of *BRCA1/2*-mutated cancer (2, 3). Other mechanisms whereby PARPi induce cell death include regulation of fork reversal and non-homologous end joining (NHEJ) at collapsed forks (4). It is also thought that inhibition of PARP activity causes a delay in single-strand breaks, which will accumulate and become toxic double-strand breaks upon encounters with the replication fork (5). PARPi are the first successful example of therapy exploiting synthetic lethality in cancer. They showed survival benefit across multiple cancers with *BRCA* mutations (6, 7).

Despite the substantial impact that PARPi have made in the clinic, most patients with metastatic disease do eventually develop resistance, creating a major unmet medical need. For instance, the SOLO2 phase III trial exemplified how 78% of *BRCA*-mutated patients with relapsed ovarian cancer eventually experienced disease progression on Olaparib, indicating the development of resistance to PARPi (8). Another example is the ARIEL2 study, which showed that 60% of *BRCA*-mutated, high-grade ovarian carcinoma patients treated with Rucaparib ultimately experienced disease progression (9). Patients that become resistant to PARPi have poor outcome and develop cross-resistance with other DNA damage agents such as platinum (10, 11).

There are various described mechanisms to render cancer cells resistance to PARPi. The first is the restoration of the homologous recombination pathway, either through reversion mutations that restore activity to the BRCA proteins (12, 13) or via loss of 53BP1 and other resection-associated proteins (14), which will, in turn, restore the homologous recombination capacity of the cell (15). Recent analyses reported that up to 80% of prostate cancer patients with *BRCA2* mutations who developed resistance to PARPi had undergone reversion mutations (16). Mutations in PARP itself can also lead to resistance to inhibitors by reducing the binding of the drug (17). Finally, loss of Poly(ADP-ribose) Glycohydrolase results in defective removal of PAR chains, potentially conferring resistance to PARPi (18).

It is expected that this resistance issue will affect approximately 40-70% of metastatic patients with *BRCA* mutations (19). Strategies aiming to combine PARPi with other drugs to overcome the hurdle of resistance have not yet proven to be successful. Strategies aiming to combine different PARPi with various chemotherapeutic drugs, such as PI3K inhibitors (20), ATR inhibitors (21, 22) or Polθ inhibitors (23, 24) have been explored but are yet to deliver impactful results with manageable toxicities. This illustrates the major need for strategies to overcome resistance to PARPi. Recently, it was shown that *BRCA*-defective cells are sensitive to treatment with the thymidine analogue CldU either alone or in combination with PARPi olaparib (5). In this study, we found that the thymidine analogue CldU conferred specific sensitivity to PARPi in *BRCA2* mutated cell lines that were previously resistant, including those with reversion mutations. We show that this combination of treatments induced high levels of DNA damage in PARP inhibitor-resistant cell lines.

## 2 Material and methods

### 2.1 Cell lines

PEO1 and PEO4 serous ovarian cancer cell lines were purchased from Sigma-Aldrich. They are derived from peritoneal ascites of the same patient with a poorly differentiated serous ovarian adenocarcinoma. PEO1 cells were collected from the patient at first relapse (cisplatin-sensitive). PEO4 cells were collected after the patient demonstrated resistance to cisplatin (25). PEO1 has *BRCA2* non-sense mutation (5193C>G, Y1655X) and PEO4 harbors *BRCA2* reversion mutation (5193C>T, Y1655Y). C4–02 and C4–13 clones were derived *in vitro* from PEO1 cells through continuous exposure to cisplatin for 4 weeks (25). C4–02 exhibited *BRCA2* reversion mutation (5192A>T). PEO1 and its 3 clones were cultured in RPMI1640 medium (+) l-glutamine supplemented with 2mM Sodium Pyruvate and 10% fetal bovine serum (FBS).

CAPAN-1 is a *BRCA2* mutant (6174delT) pancreatic cancer cell line. Its clones C2-5, C2–8 and C2–13 were derived *in vitro* through continuous exposure to cisplatin for 4 weeks. C2–5 exhibited *BRCA2* reversion mutation (6006_6308del303) while C2–08 and C2–13 do not have reversion mutations (13). CAPAN-1 and its 3 clones were cultured in RPMI1640 medium (+) l-glutamine supplemented with 2mM Sodium Pyruvate and 10% fetal bovine serum (FBS). PEO1 derived clones (C4–02 and C4-13), CAPAN-1 and its clones (C2-05, C2–08 and C2-13) were generously provided by Prof. Toshiyasu Taniguchi (Tokai University school of medicine).

### 2.2 Drugs and chemicals

Olaparib (HY-10162), CldU (Merck, C6891) and Saruparib (HY-132167) were purchased from MedChemExpress (LUCERNA-CHEM). Thymidine (T1895) was purchased from Sigma-Aldrich, EdU (A10044) from ThermoFisher Scientific and BrdU (B23151 from Invitrogen). The stock solutions of PARPi and chemical compounds were prepared from powders dissolved in 100% dimethyl sulfoxide (DMSO) for a stock solution concentration of 10mM except for thymidine that was dissolved in water, aliquoted, and stored at −80°C for up to a maximum of 12 months. In order to minimize the cytotoxic effect of DMSO dilution solution on the cells, several intermediate dilutions were prepared to dispense 2µL of inhibitors in 2mL medium per well of a 6-well plate. The same volume of DMSO was added to control wells.

### 2.3 Clonogenic assay

The cytotoxic activity of drugs and their influence on cell growth, survival and their ability to form colonies were assessed using the colony formation assay. Briefly, cells were seeded in 6-well plates in 2 mL of culture medium in triplicate (1500 cells per well for CAPAN-1 and its clones, and 3000 cells for PEO1 and its clones) and incubated for 24 h (37°C, 5% CO2). Drugs were added to the medium 24h after cell seeding with pre-selected doses of tested compounds (0.001 – 10μM olaparib, 10 - 100–1000 nM saruparib, 0.05 - 5 μM CldU or their combinations) by adding 2μL of 1000 × concentrated drugs prepared in DMSO. The same volume of DMSO was added to control wells. After 48h, the medium was changed, and cells were allowed to grow and proliferate in a drug-free medium for 14–21 days until non-overlapping colonies were formed in control wells. Colonies were fixed with paraformaldehyde (PFA) 4% for 20 min, stained with 0.5% crystal violet in 20% ethanol for 20 min, thoroughly rinsed with deionized water to remove residual dye, and air-dried at room temperature. Each well was photographed using the FUSION FX6 EDGE Imaging System and number of colonies was quantified using ImageJ software^®^ with colony counting extension. A colony of at least a size of 20 pixel^2^ was scored as one survivable colony and considered for the count. Results were expressed as relative survival (percentage of colonies) as the number of colonies per treatment versus colonies that appeared in the DMSO control (mean colony counts ± standard errors are reported). Graphs were generated using GraphPad Prism^®^, 9 software (v.9.4.1).

### 2.4 Flow cytometry

Following drug treatment, cells were harvested by trypsin and fixed in 70% ethanol in PBS1X overnight at −20°C. Detection of γH2AX phosphorylation was performed using the Guava Histone H2AX Phosphorylation Assay Kit (Luminex, catalogue no. FCCS100182) according to the manufacturer’s instructions. Genomic DNA was stained by incubating the cells in PBS containing RNase (Roche, catalogue no. 11119915001) and propidium iodide (Sigma-Aldrich catalogue no. 81845). DNA-γH2AX profiles were acquired by flow cytometry (CytoFLEX LX flow cytometer); more than 5,000 cells were analyzed per sample using Kaluza^®^ software (Beckman Coulter).

### 2.5 Statistical analysis

Statistical analysis was performed using GraphPad Prism 9 software (v.9.4.1). Detailed description of means or medians, error bars and the number replicates and/or cells analyzed is reported in the figure legends. For comparison of more than two groups, the two-way ANOVA with Tukey’s multiple comparisons test was used Values are presented as mean ± SEM. p<0.05 was considered significant. Detailed description of means or medians, error bars and the number replicates and/or cells analyzed is reported in the figure legends. Statistical analysis was reported on **Supplementary Tables**.

## 3 Results

### 3.1 BRCA2-mutant cells’ sensitivity to CldU resembles the sensitivity to PARP inhibitor

**Figure 1:**
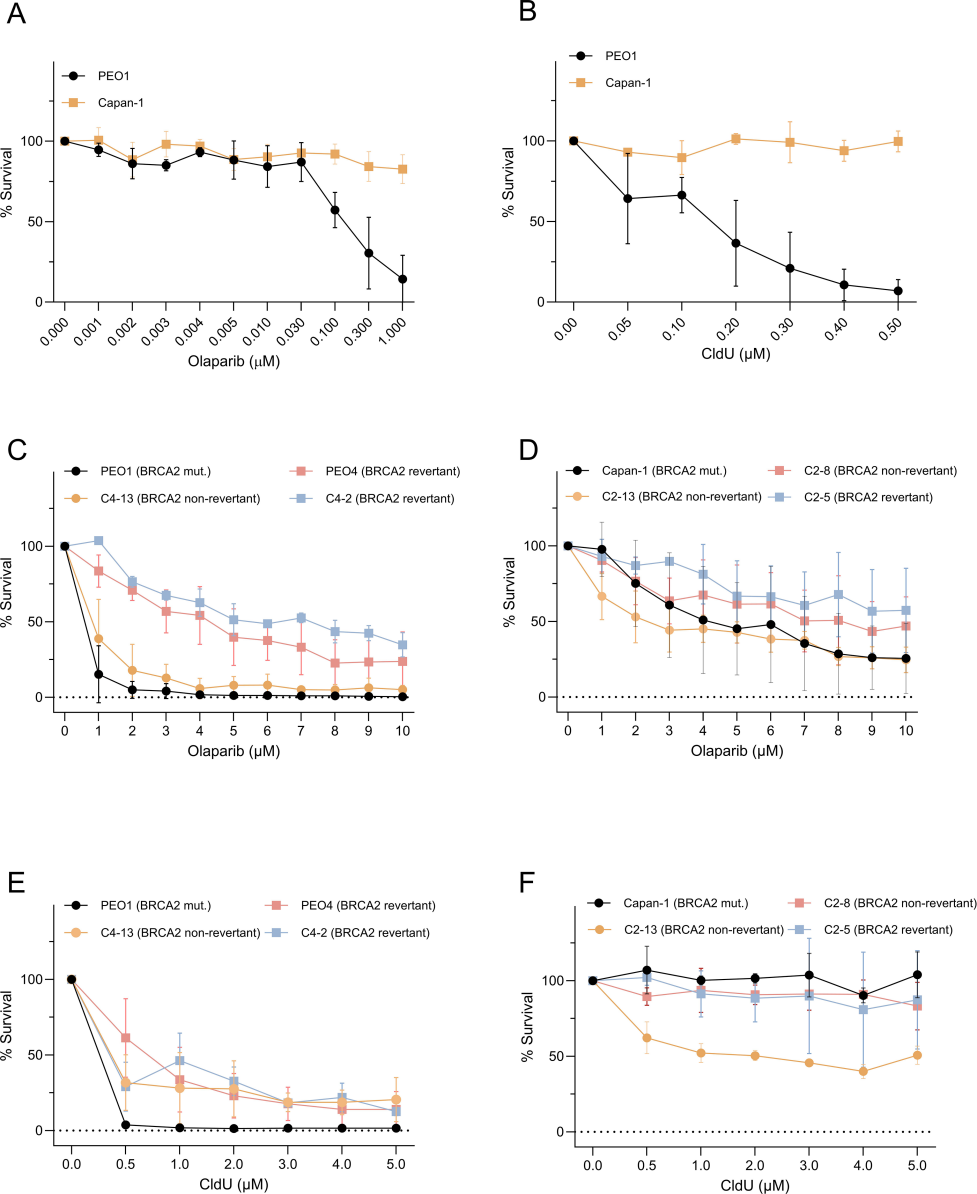
Clonogenic sensitivity to olaparib and CldU in BRCA2-mutant cancer cells and their PARPi-resistant derivatives. **(A, B)** Dose–response of BRCA2-deficient PEO1 and Capan-1 cells treated for 48 h with increasing concentrations of **(A)** the PARP inhibitor olaparib (0.0001–1 µM) or **(B)** chlorodeoxyuridine (CldU; 0.05–0.5 µM). Survival is expressed as percent of untreated control. **(C, D)** Olaparib sensitivity in BRCA2-mutant parental lines versus isogenic PARPi-resistant clones. **(C)** PEO1 (BRCA2-mutant) compared to C4-13 (non-revertant resistant) and two BRCA2-reversion derivatives (PEO4, C4-2). **(D)** Capan-1 (BRCA2-mutant) compared to C2-13 (non-revertant) and two BRCA2-reversion clones (C2-8, C2-5). **(E, F)** Corresponding clonogenic survival following 48 h CldU treatment in the same sets of PEO1-derived [**(E)** PEO1, PEO4, C4-2, C4-13] and Capan-1-derived [**(F)** Capan-1, C2-8, C2-13, C2-5] cell lines. In all panels, data are mean ± SD of three independent biological replicates; curves are normalized to untreated controls.

Recent findings have demonstrated that BRCA1-deficient cells exhibit marked sensitivity to chlorodeoxyuridine (CldU), both as a monotherapy and in combination with the PARP inhibitor olaparib (4). In this study, we assessed the sensitivity to CldU across eight *BRCA2*-mutant cancer cell lines. These included: (1) PEO1, an ovarian cancer-derived cell line, and its isogenic derivatives resistant to cisplatin, either with (PEO4; C4-02) or without *BRCA2* reversion mutation (25); and (2) CAPAN-1, a pancreatic cancer-derived cell line, along with its cisplatin-resistant clones due to either *BRCA2* reversion mutations (C2–05 and C2-13) or other mechanisms (C2-08) (13). Our results revealed that sensitivity to CldU partially reflected sensitivity to PARP inhibitor olaparib. Notably, PEO1 displayed pronounced sensitivity to olaparib (**Figures 1A–C**) and CldU (**Figure 1E**), whereas PEO4, C4-02, CAPAN-1, C2-05, C2-08, and C2–13 exhibited reduced sensitivity to both olaparib (**Figures 1C, D**) and CldU (**Figures 1E, F**).

### 3.2 CldU sensitizes PARP inhibitor-resistant cells to PARP inhibitors

**Figure 2:**
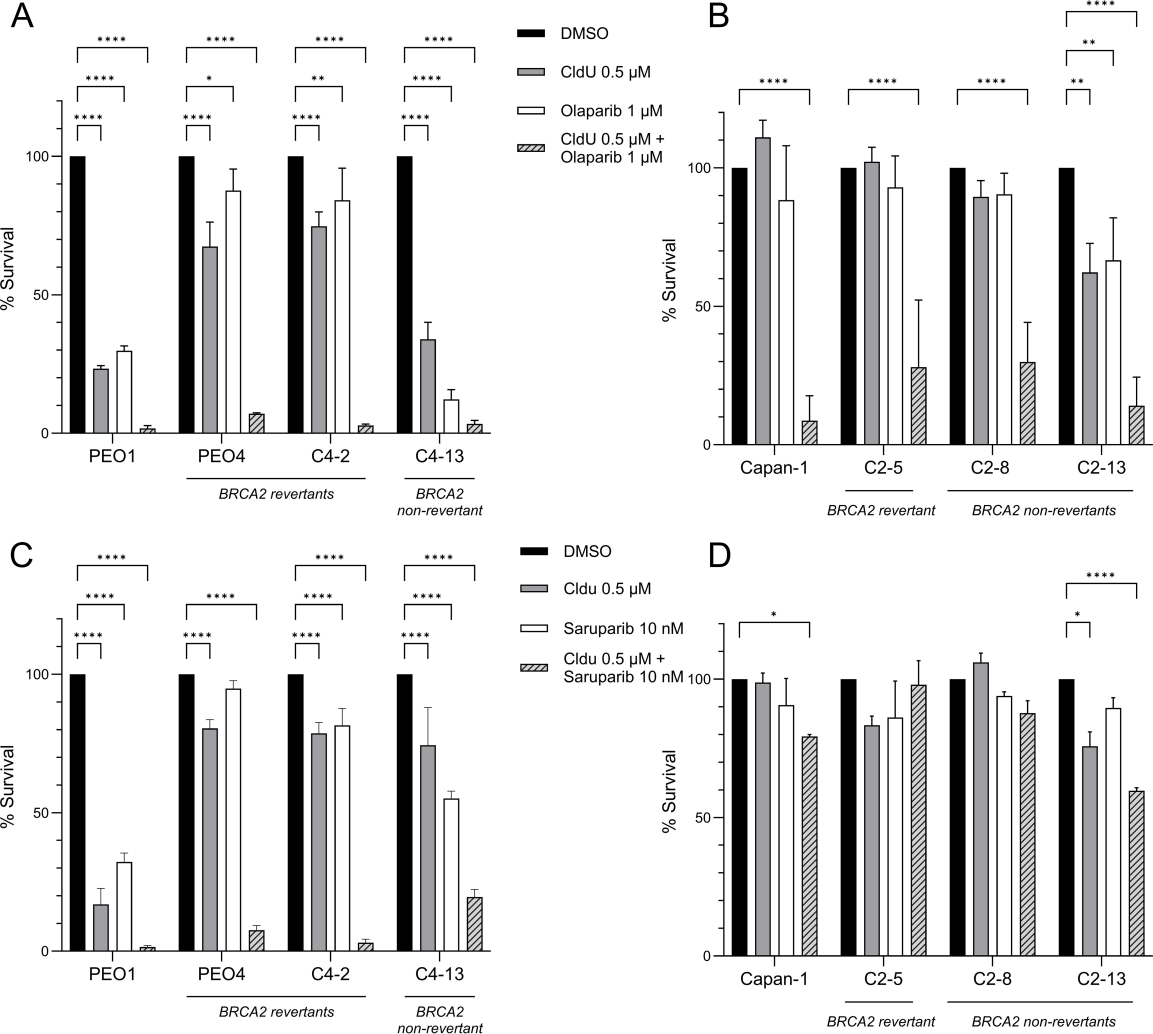
Synergistic cytotoxicity of CldU and PARP inhibitors in BRCA2-deficient and PARPi-resistant cell lines. **(A, B)** Clonogenic survival of parental BRCA2-mutant cells and their isogenic PARPi-resistant derivatives after 48 h treatment with vehicle (DMSO), CldU (0.5 µM), olaparib (1 µM), or the combination. **(A)** PEO1 (BRCA2-mutant), PEO4 and C4-2 (BRCA2-revertant resistant), and C4-13 (non-revertant resistant). Data are mean ± SD of three technical replicates from one representative experiment (n = 3 independent repeats). **(B)** Capan-1 (BRCA2-mutant), C2–5 and C2-8 (BRCA2-revertant resistant), and C2-13 (non-revertant resistant). Data are mean ± SD of three independent biological replicates. **(C, D)** Clonogenic survival of the same cell panels treated for 48 h with CldU (0.5 µM), the PARP-1 selective inhibitor saruparib (10 nM), or their combination. **(C)** PEO1 lineage (PEO1, PEO4, C4-2, C4-13); mean ± SD of three technical replicates from one representative experiment (n = 3). **(D)** Capan-1 lineage (Capan-1, C2-5, C2-8, C2-13); mean ± SD of three technical replicates from one representative experiment (n=3). Statistical significance was assessed using GraphPad Prism 10.5.0 software by two-way ANOVA with Tukey’s multiple comparisons test. *P < 0.05, **P < 0.01, ***P < 0.001, ****P < 0.0001. In all panels, the striped bars (combination) reveal pronounced loss of clonogenic survival in both parental and PARPi-resistant clones, indicating strong synergy between CldU and either olaparib or saruparib.

We next investigated whether the combination of CldU and PARPi exerts a synergistic effect in *BRCA2*-mutant cancer cells. Remarkably, the co-treatment with low doses of olaparib (1 μM) and CldU (0.5 μM) proved to be lethal in *BRCA2*-mutant PEO1 cells, as well as in its olaparib-resistant isogenic derivatives, including revertant clones PEO4 and C4-02 (**Figure 2A**, **Supplementary Table 1** and **Supplementary Figure 1A**). This synergistic effect was further validated using saruparib (AZ5305), a second-generation, highly potent and PARP1-selective inhibitor with approximately 500-fold selectivity for PARP1 over PARP2 (18). Low-dose saruparib (10 nM) combined with CldU resulted in >80% cell death across the three PEO1-derived clones, all of which were resistant to saruparib monotherapy (**Figure 2C**, **Supplementary Table 3**, **Supplementary Figure 1C** and **Supplementary Figures 2A–D**). Consistently, the combination of CldU with olaparib (**Figure 2B**, **Supplementary Table 2** and **Supplementary Figure 1B**) elicited a synergistic response in the *BRCA2*-mutant CAPAN-1 cell line and its PARP inhibitor-resistant isogenic derivatives, including the reversion-bearing C2–05 clone. Interestingly, the synergistic response between saruparib and CldU in CAPAN-1 cells was less significant (**Figure 2D**, **Supplementary Table 4**, **Supplementary Figure 1D** and **Supplementary Figures 2E–H**). This could reflect the high intrinsic resistance of these cells to both agents, in addition to saruparib being a PARP1 specific inhibitor with lower trapping potential than olaparib (26). Collectively, these findings demonstrate that CldU and PARPi act synergistically in *BRCA2*-mutant cancer cells, even in the context of acquired PARP inhibitor resistance, including resistance mediated by *BRCA2* reversion mutations.

### 3.3 The synergistic effect of CldU and PARP inhibitor is specific

**Figure 3:**
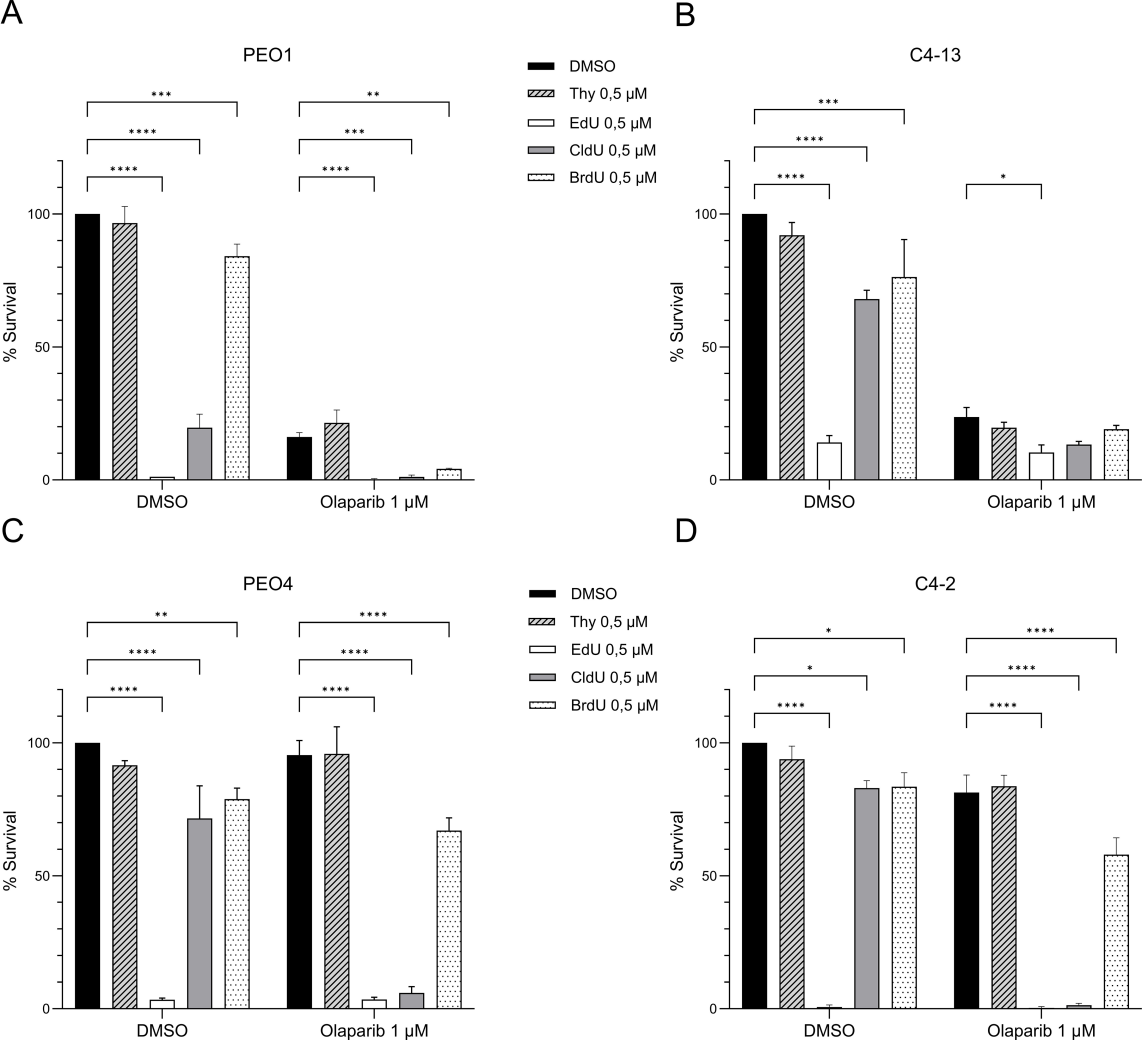
CldU is the most potent and selective thymidine analogue for synergizing with PARP inhibition. **(A–D)** Clonogenic survival of BRCA2-mutant PEO1 cells and isogenic derivatives following 48-hour treatment with thymidine analogues (Thymidine, EdU, CldU, or BrdU; 0.5 µM) alone or combined with olaparib (1 µM). **(A)** PEO1 parental cells. **(B)** C4-13 (PARPi-resistant, BRCA2 non-revertant). **(C)** PEO4 (PARPi-resistant, BRCA2-revertant). **(D)** C4-2 (PARPi-resistant, BRCA2-revertant). Data represent the mean ± SD of three technical replicates from one representative experiment (n=3). Statistical significance was assessed using GraphPad Prism 10.5.0 software by two-way ANOVA with Tukey’s multiple comparisons test. *P < 0.05, **P < 0.01, ***P < 0.001, ****P < 0.0001. Experiments were repeated independently three times for panels **(A, C)**, and twice for panels **(B, D)**.

CldU is a thymidine analogue with a chemical structure closely resembling that of native thymidine. It is commonly used in molecular biology to label newly synthesized DNA, as it is incorporated into DNA but not RNA. Other thymidine analogues, such as 5-ethynyl-2′-deoxyuridine (EdU) and 5-bromo-2′-deoxyuridine (BrdU), serve similar roles in tracking DNA synthesis (**Figure 3A**). To determine whether the observed synergy between CldU and PARPi is unique to CldU or shared among thymidine analogues, we evaluated the cytotoxic effects of olaparib (1 μM) in combination with thymidine or its analogues (CldU, BrdU, and EdU) at an equivalent concentration (0.5 μM) in PEO1 and PEO4 cell lines. Our results demonstrated that the synergistic interaction with olaparib was specific to CldU (**Figures 3B-E** and **Supplementary Figure 3**). In contrast, EdU exhibited intrinsic cytotoxicity across all conditions, independent of olaparib co-treatment (**Figures 3B–E** and **Supplementary Figure 3**). This result is consistent with a recent report showing that EdU induces DNA damage in mammalian cells, that is repaired by nucleotide excision repair (27). Neither thymidine nor BrdU alone, nor in combination with olaparib, exhibited significant cytotoxic effects. These findings suggest that the synergy between CldU and PARPi is not a general property of thymidine analogues, but rather a specific feature of CldU.

### 3.4 CldU combination with PARP inhibitor induce DNA damage

**Figure 4:**
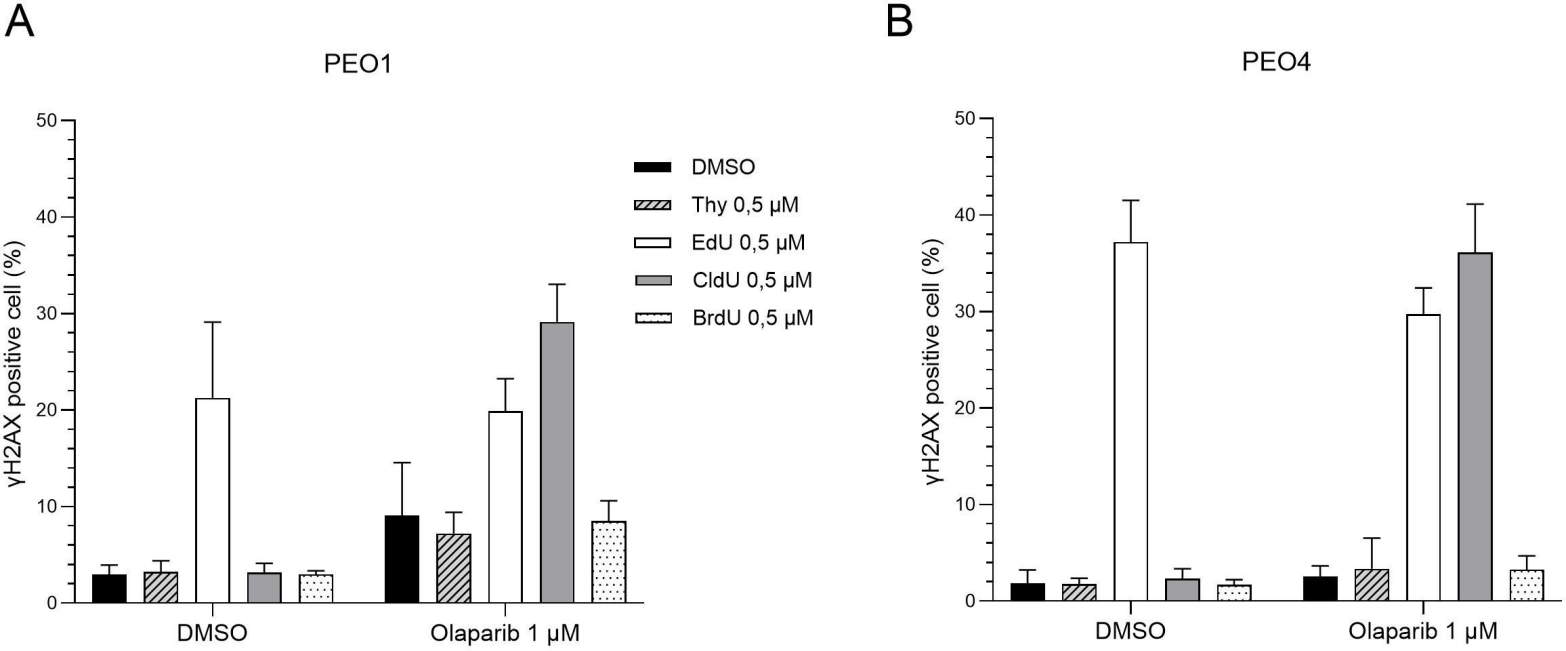
The chlorine group in CldU drives enhanced DNA damage in BRCA2-mutant cells under PARP inhibition. Quantification of γH2AX-positive cells (marker of DNA damage) by flow cytometry after 48-hour treatment with thymidine analogues (Thymidine, EdU, CldU, or BrdU; 0.5 µM), alone or combined with olaparib (1 µM). **(A)** PEO1 (BRCA2-mutant parental line). **(B)** PEO4 (PARPi-resistant, BRCA2-revertant). Data show the percentage of γH2AX-positive cells from three independent experiments. Increased DNA damage in CldU-treated groups highlights the role of the chlorine modification under PARP inhibition.

Finally, we sought to determine whether the combination of CldU and PARP inhibition induces DNA damage in *BRCA2*-mutant cancer cells. As expected, treatment with olaparib alone triggered DNA damage in PARP-sensitive PEO1 cells (**Figure 4A**). In contrast, olaparib monotherapy did not elicit substantial DNA damage in PARP-resistant PEO4 and C4–02 cells (**Figure 4B** and **Supplementary Figure 4**). Notably, co-treatment with CldU and olaparib resulted in marked DNA damage in these resistant cell lines (**Supplementary Figure 4**). Furthermore, the combination of CldU and olaparib induced early S-phase cell cycle arrest in both PEO1 and PEO4 cells (**Supplementary Figure 4**), consistent with replication stress-associated DNA damage. In parallel, EdU treatment led to DNA damage across all conditions (**Figures 4A, B**), independent of *BRCA2* status, underscoring its inherent cytotoxicity. Collectively, these findings demonstrate that CldU and olaparib cooperate to induce DNA damage in PARPi-resistant cells, supporting a synergistic mechanism of action.

## 4 Discussion

PARP inhibitors (PARPi) have significantly advanced the treatment of cancers harboring *BRCA1* or *BRCA2* mutations by exploiting deficiencies in homologous recombination-mediated DNA repair. However, resistance to PARPi remains a major clinical challenge. Reversion mutations in *BRCA1/BRCA2*—observed in up to 80% of patients who develop resistance to PARPi—can restore protein function, thereby reinstating DNA repair capability and leading to therapeutic resistance and poor outcomes (16, 17). Strategies to overcome PARPi resistance are actively being explored. In this study, we demonstrate that the thymidine analogue chlorodeoxyuridine (CldU) sensitizes PARPi-resistant cancer cells to PARP inhibition. This cytotoxic effect is thought to result from the accumulation of single-stranded DNA gaps initiated by uracil DNA glycosylase-mediated base excision repair. When combined with PARPi-induced replication stress and compromised fork protection in *BRCA*-deficient cells, this leads to lethal levels of DNA damage. Notably, even cells harboring *BRCA* reversion mutations, which partially restore homologous recombination, remain sensitive to the combination of CldU and PARPi. This suggests that the mechanism of cytotoxicity may bypass conventional *BRCA*-mediated repair pathways. Although the exact mechanism of cell death remains to be fully elucidated, our findings point to a potentially novel vulnerability in PARPi-resistant cancers. Of note, we observed that the combination of CldU and olaparib was synergetic across all cell lines derived from both PEO1 and CAPAN-1, while the synergistic effect between saruparib and CldU in CAPAN-1 cells was less significant. Elucidating whether this is due to intrinsic differences in DNA repair between cell lines, replication stress response, or PARP trapping efficiency (26) need to be addressed in the future.

Importantly, while CldU is not approved for clinical use and is currently limited to research applications as a DNA synthesis marker, clinically approved nucleoside analogues such as gemcitabine, cytarabine, and trifluridine share structural similarities. Some of these, particularly gemcitabine, have shown synergistic activity with PARPi in preclinical models of non-small-cell lung cancer (28) and in a clinical trial that enrolled pancreatic cancer patients (29). Next-generation antibody drug conjugates combining dual payloads that target DNA damage, for instance topoisomerase 1 inhibitor and PARPi, are currently investigated (30) and could be a therapeutic approach to reduce the toxicities of such combinations. Our work also confirmed that another thymidine analogue, EdU, is cytotoxic and induces DNA damage in mammalian cancer cells (27, 31), independent of *BRCA2* status. Overall, our findings prompt further investigation into nucleotide analogues for the treatment of PARPi-resistant cancers.

## Data Availability

The raw data supporting the conclusions of this article will be made available by the authors, without undue reservation.

## Supplementary material

Supplementary Figure 1 Representative colony formation assays showing the effects of combined CldU and PARP inhibition on BRCA2-mutant and PARPi-resistant cell lines. **(A, B)** Representative images from clonogenic survival assays following 48-hour treatment with olaparib (1 µM) plus CldU (0.5 µM) in: **(A)** PEO1 (BRCA2-mutant) and its isogenic derivatives (PEO4, C4-2, C4-13) **(B)** Capan-1 (BRCA2-mutant) and its isogenic derivatives (C2-8, C2-13, C2-5) **(C, D)** Representative images from clonogenic survival assays following 48-hour treatment with saruparib (10 nM) plus CldU (0.5 µM) in: **(C)** PEO1 and its isogenic derivatives (PEO4, C4-2, C4-13) **(D)** Capan-1 and its isogenic derivatives (C2-8, C2-13, C2-5). For each condition, one well from triplicate experiments is shown.

Supplementary Figure 2 Effect of saruparib and CldU combination treatment on clonogenic survival in BRCA2-mutant and PARPi-resistant cell lines. Clonogenic survival of BRCA2-mutant PEO1 and Capan-1 cells, and their isogenic derivatives (PEO4, C4-2, C4-13, C2-5, C2-8, C2-13), after 48-hour treatment with saruparib alone (10 nM, 100 nM, or 1 µM) or in combination with CldU (0.5 µM). Data represent one experiment with a single well per treatment condition.

Supplementary Figure 3 Representative colony formation assays showing the effects of thymidine analogues combined with PARP inhibition. **(A)** Chemical structures of the thymidine analogues used in this study (sourced from PubChem, public domain). **(B–E)** Representative images from clonogenic survival assays following 48-hour treatment with thymidine analogues (Thymidine, EdU, CldU, or BrdU; 0.5 µM), alone or in combination with olaparib (1 µM), in: **(A)** PEO1 (BRCA2-mutant parental line) **(B)** C4-13 (PARPi-resistant, BRCA2 non-revertant) **(C)** PEO4 (PARPi-resistant, BRCA2-revertant) (D) C4-2 (PARPi-resistant, BRCA2-revertant). For each condition, one well from triplicate experiments is shown.

Supplementary Figure 4 Representative flow cytometry analysis of cell cycle distribution and DNA damage following treatment with thymidine analogues and PARP inhibition. **(A–D)** Representative flow cytometry plots showing cell cycle distribution and γH2AX staining after 48-hour treatment with thymidine analogues (Thymidine, EdU, CldU, or BrdU; 0.5 µM), alone or combined with olaparib (1 µM), in: **(A)** PEO1 (BRCA2-mutant parental line) **(B)** PEO4 (PARPi-resistant, BRCA2-revertant) **(C)** C4.13 (PARPi resistant, BRCA2 non-revertant) **(D)** C4.2 (PARPi resistant, BRCA2-revertant) Data represent the percentage of γH2AX-positive cells from one representative independent experiment.

Supplementary Table 1 statistical tests of **Figure 2A**. Test used: Two-way ANOVA with Tukey’s multiple comparisons test.

Supplementary Table 2 statistical tests of **Figure 2B**. Test used: Two-way ANOVA with Tukey’s multiple comparisons test.

Supplementary Table 3 statistical tests of **Figure 2C**. Test used: Two-way ANOVA with Tukey’s multiple comparisons test.

Supplementary Table 4 statistical tests of **Figure 2D**. Test used: Two-way ANOVA with Tukey’s multiple comparisons test.
