# Supplementary figures and images for: CldU sensitizes BRCA2 reverse-mutated cells to PARP inhibitors
Source: Front Oncol. 2025 Nov 19;15:1626301. doi: 10.3389/fonc.2025.1626301 (PMC12672267; doi:10.3389/fonc.2025.1626301)

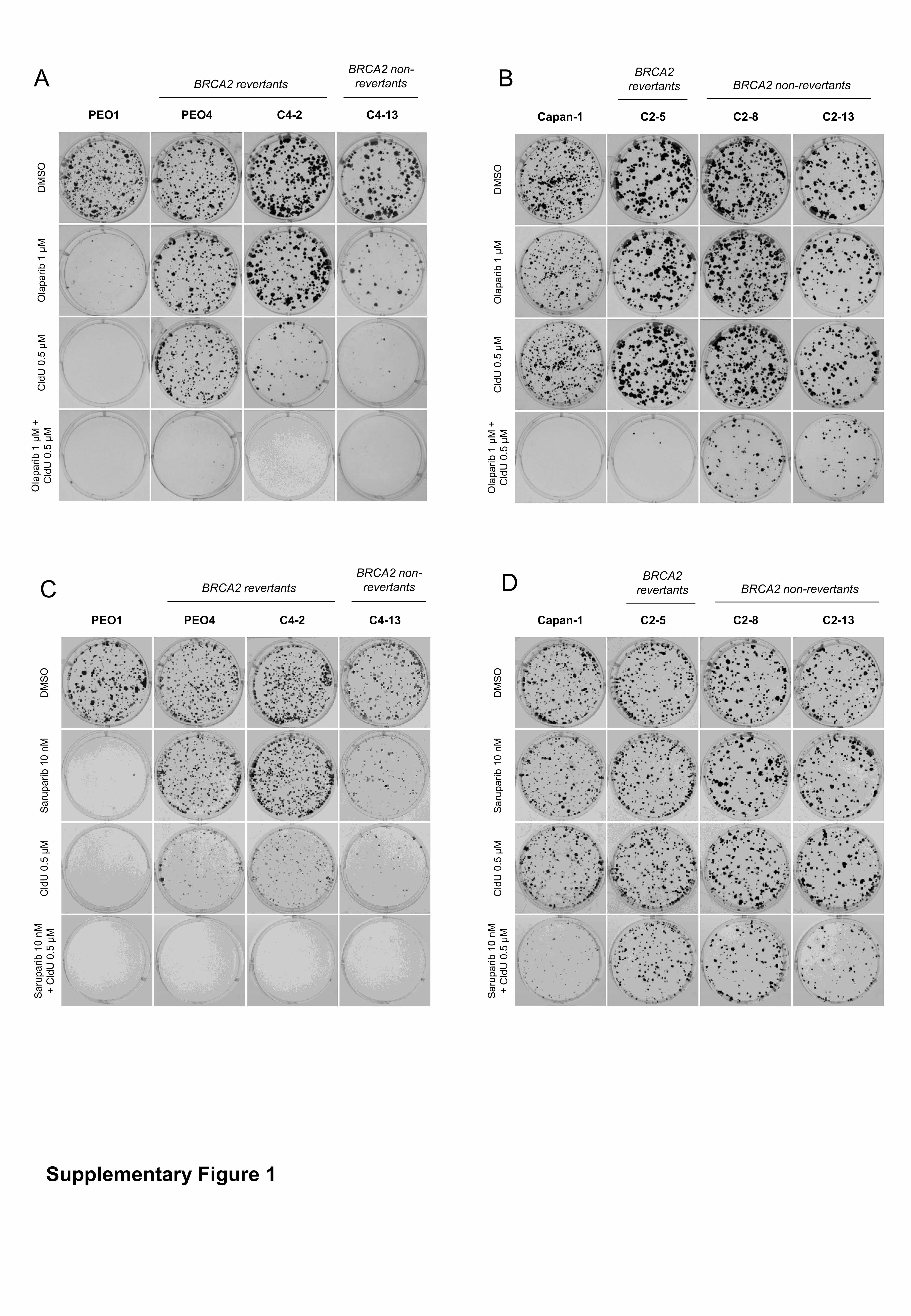

Supplement: Supplementary Figure 1 — Representative colony formation assays showing the effects of combined CldU and PARP inhibition on BRCA2-mutant and PARPi-resistant cell lines. (A, B) Representative images from clonogenic survival assays following 48-hour treatment with olaparib (1 µM) plus CldU (0.5 µM) in: (A) PEO1 (BRCA2-mutant) and its isogenic derivatives (PEO4, C4-2, C4-13) (B) Capan-1 (BRCA2-mutant) and its isogenic derivatives (C2-8, C2-13, C2-5) (C, D) Representative images from clonogenic survival assays following 48-hour treatment with saruparib (10 nM) plus CldU (0.5 µM) in: (C) PEO1 and its isogenic derivatives (PEO4, C4-2, C4-13) (D) Capan-1 and its isogenic derivatives (C2-8, C2-13, C2-5). For each condition, one well from triplicate experiments is shown. [file Image1.jpeg]

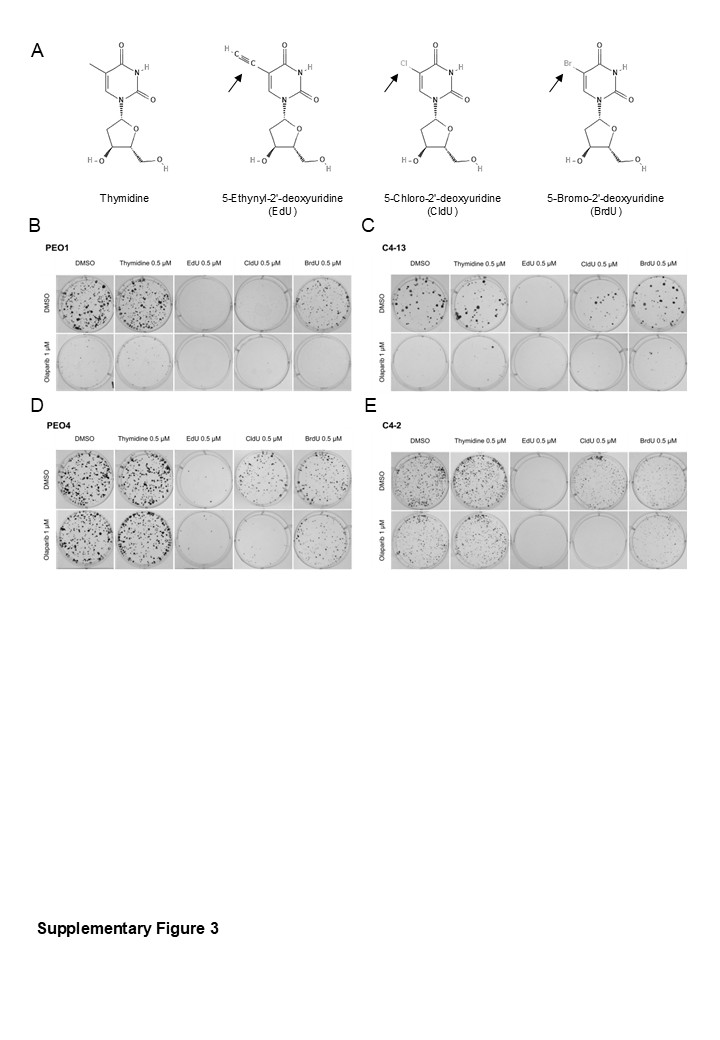

Supplement: Supplementary Figure 3 — Representative colony formation assays showing the effects of thymidine analogues combined with PARP inhibition. (A) Chemical structures of the thymidine analogues used in this study (sourced from PubChem, public domain). (B–E) Representative images from clonogenic survival assays following 48-hour treatment with thymidine analogues (Thymidine, EdU, CldU, or BrdU; 0.5 µM), alone or in combination with olaparib (1 µM), in: (A) PEO1 (BRCA2-mutant parental line) (B) C4-13 (PARPi-resistant, BRCA2 non-revertant) (C) PEO4 (PARPi-resistant, BRCA2-revertant) (D) C4-2 (PARPi-resistant, BRCA2-revertant). For each condition, one well from triplicate experiments is shown. [file Image3.jpg]

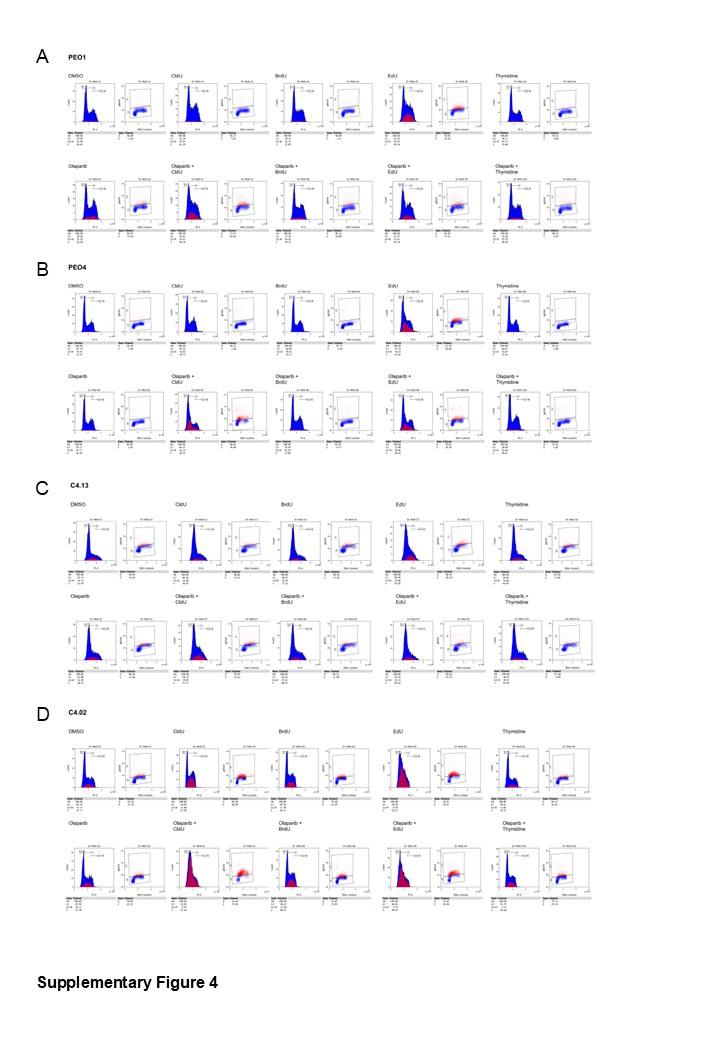

Supplement: Supplementary Figure 4 — Representative flow cytometry analysis of cell cycle distribution and DNA damage following treatment with thymidine analogues and PARP inhibition. (A–D) Representative flow cytometry plots showing cell cycle distribution and γH2AX staining after 48-hour treatment with thymidine analogues (Thymidine, EdU, CldU, or BrdU; 0.5 µM), alone or combined with olaparib (1 µM), in: (A) PEO1 (BRCA2-mutant parental line) (B) PEO4 (PARPi-resistant, BRCA2-revertant) (C) C4.13 (PARPi resistant, BRCA2 non-revertant) (D) C4.2 (PARPi resistant, BRCA2-revertant) Data represent the percentage of γH2AX-positive cells from one representative independent experiment. [file Image4.jpg]

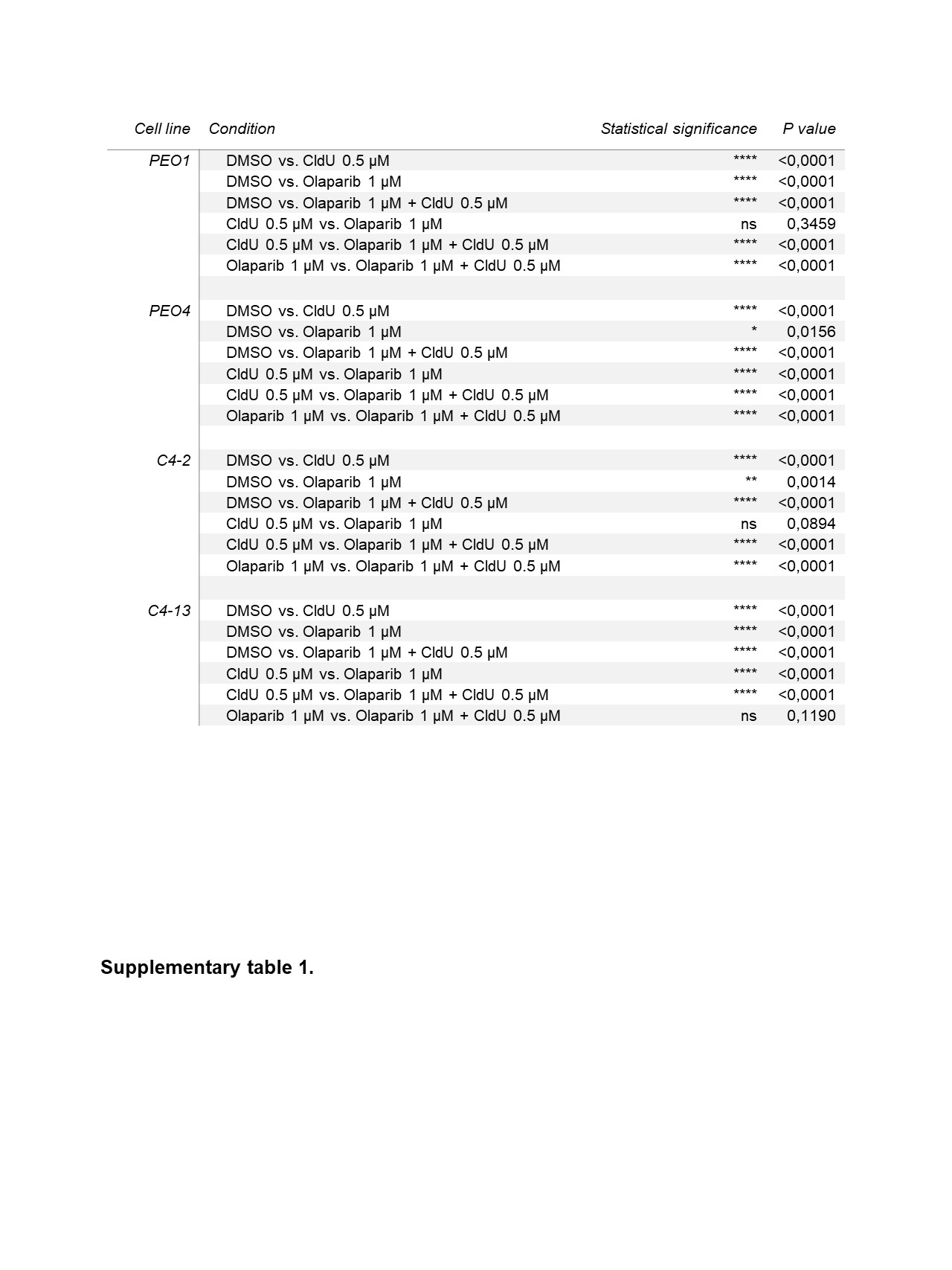

Supplement: Supplementary Table 1 — statistical tests of Figure 2A. Test used: Two-way ANOVA with Tukey’s multiple comparisons test. [file DataSheet1.zip › Supplementary Table 1.JPEG]

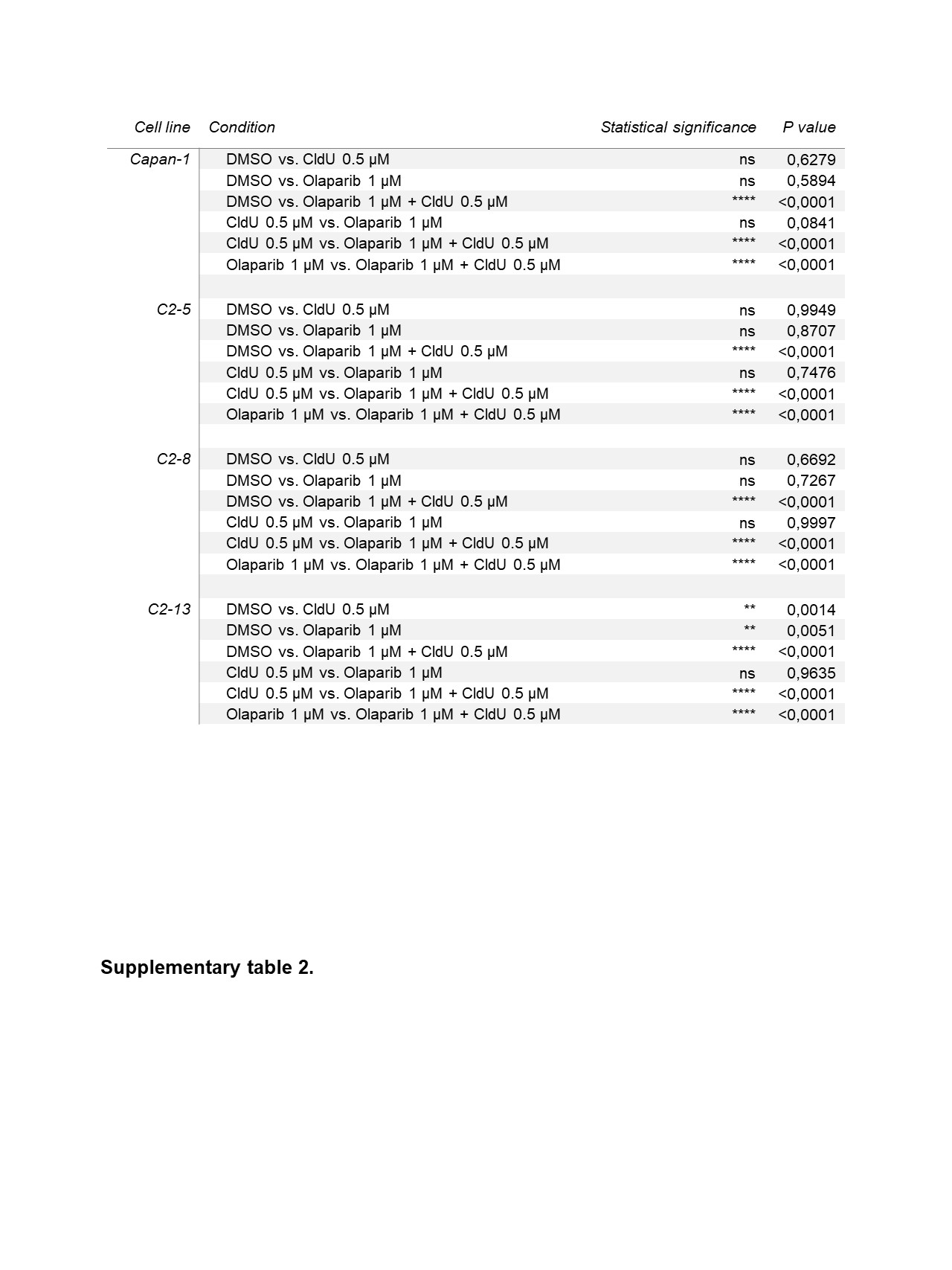

Supplement: Supplementary Table 1 — statistical tests of Figure 2A. Test used: Two-way ANOVA with Tukey’s multiple comparisons test. [file DataSheet1.zip › Supplementary Table 2.JPEG]

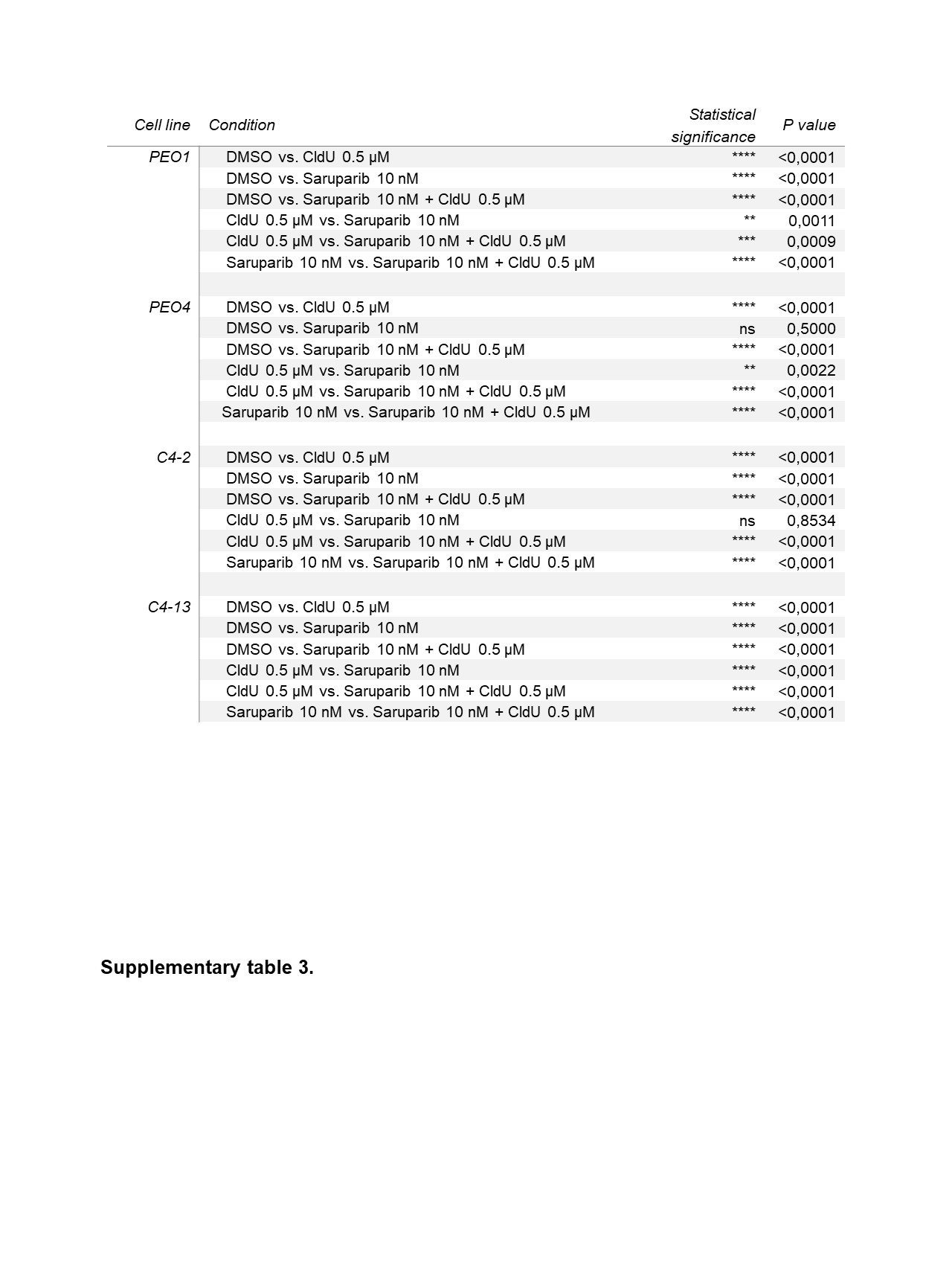

Supplement: Supplementary Table 1 — statistical tests of Figure 2A. Test used: Two-way ANOVA with Tukey’s multiple comparisons test. [file DataSheet1.zip › Supplementary Table 3.JPEG]

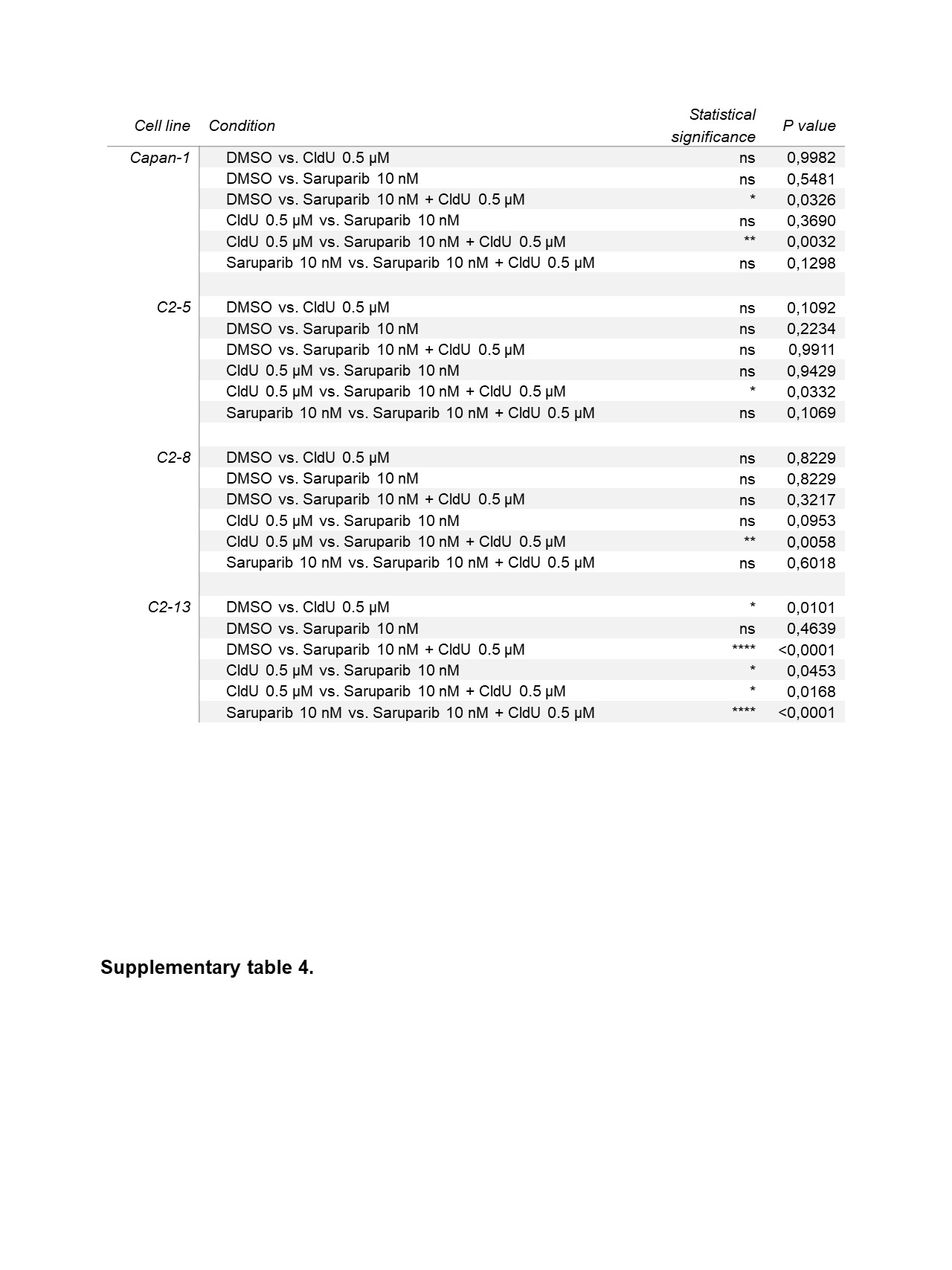

Supplement: Supplementary Table 1 — statistical tests of Figure 2A. Test used: Two-way ANOVA with Tukey’s multiple comparisons test. [file DataSheet1.zip › Supplementary Table 4.JPEG]
